# Supplementary material for: Outcome of Fetal Dysrhythmias with and without Extracardiac Anomalies
Source: Diagnostics (Basel). 2023 Jan 29;13(3):489. doi: 10.3390/diagnostics13030489 (PMC9914765; doi:10.3390/diagnostics13030489)
Supplement: Supplementary file 1 [file diagnostics-13-00489-s001.zip › diagnostics-2141845-supplementary.pdf]

Table S1. Characteristics of neonates with neonatal death

Nr	type of arrhythmia	GA at diagnosis	CHD	ECA	intrauterine treatment	GA at birth	APGAR	birthweight	sex	survivaltime (days)	cause of death
1	Bradyarrhythmia	21+0	HLHS, Rhabdomyoma	0	0	38+6	5/7/7	2965	f	125	Heart failure
2	Tachyarrhythmia	27+6	0	0	Digoxin,Flecanoide	28+5	7/7/8	1390	m	2	Neonatal sepsis
3	Tachyarrhythmia	29+4	0	Analatresia	Digoxin	30+1	7/9/9	1760	f	185	Respiratory insufficiency
4	Rhythm irregularities	23+3	Ebstein	0	Digoxin	29+4		1865	m	13	Heart failure
5	Bradyarrhythmia	20+6	TGA,DOLV	Heterotaxia	0	34+1	3/1/0	4100	m	0	Heart failure
6	Rhythm irregularities	26+5	Ebstein	0	0	31+3	6/8/9	1470	m	7	Heart failure
7	Tachyarrhythmia	21+2	0	0	0	23+4	6/8/9	550	m	4	Heart failure
8	Bradyarrhythmia	24+1	0	0	0	25+6	8/8/9	1046	m	22	MODS
9	Tachyarrhythmia	12+5	0	0	0	29+0	1/0/0	836	m	0	Pulmonary hypoplasia
